# Preventive effect of artemisinin extract against cholestasis induced via lithocholic acid exposure

**DOI:** 10.1042/BSR20181011

**Published:** 2018-11-21

**Authors:** Adel Qlayel Alkhedaide, Tamer Ahmed Ismail, Saad Hmoud Alotaibi, Mohamed Abdo Nassan, Zafer Saad Al Shehri

**Affiliations:** 1 Medical Laboratory Department, Faculty of Applied Medical Sciences, Turabah, Taif University, Saudi Arabia; 2 Department of Physiology, Faculty of Veterinary Medicine, Zagazig University, Egypt; 3 Department of Chemistry, Faculty of Applied Medical Sciences, Turabah, Taif University, Saudi Arabia; 4 Department of Pathology, Faculty of Veterinary Medicine, Zagazig University, Egypt; 5 Clinical Laboratory Department, Al-Dawadmi College of Applied Medical Sciences, Shaqra University, Saudi Arabia

**Keywords:** Artemisinin, Bile duct, Cholestasis, hepatotoxicity, Lithocholic acid

## Abstract

Obstructive cholestasis characterized by biliary pressure increase leading to leakage of bile back that causes liver injury. The present study aims to evaluate the effects of artemisinin in obstructive cholestasis in mice. The present study was carried out on 40 adult healthy mice that were divided into 4 groups, 10 mice each; the negative control group didn’t receive any medication. The normal group was fed normally with 100 mg/kg of artemisinin extract orally. The cholestatic group fed on 1% lithocholic acid (LCA) mixed into control diet and cholestatic group co-treated with 100 mg/kg of artemisinin extract orally. Mice were treated for 1 month then killed at end of the experiment. A significant increase in alanine aminotransferase, aspartate aminotransferase, and total and direct bilirubin was detected in mice exposed to LCA toxicity. That increase was significantly reduced to normal values in mice co-treated with artemisinin. LCA toxicity causes multiple areas of necrosis of irregular distribution. However, artemisinin co-treatment showed normal hepatic architecture. Moreover, LCA causes down-regulation of hepatic mRNA expressions of a set of genes that are responsible for ATP binding cassette and anions permeability as ATP-binding cassette sub-family G member 8, organic anion-transporting polypeptide, and multidrug resistance-associated protein 2 genes that were ameliorated by artemisinin administration. Similarly, LCA toxicity significantly down-regulated hepatic mRNA expression of constitutive androstane receptor, *OATP4*, and farnesoid x receptor genes. However, artemisinin treatment showed a reasonable prevention. In conclusion, the current study strikingly revealed that artemisinin treatment can prevent severe hepatotoxicity and cholestasis that led via LCA exposure.

## Introduction

The medical definition of cholestasis is retaining of bile-excreted substances into the bile itself again. There are many different causes underlying this condition, including inherited and acquired pathologies. Inherited cholestasis is an autosomal recessive disease, while the acquired cholestasis refers to bile secretion caused via several defects such as bile duct obstruction, hepatitis, biliary cirrhosis, cholangiocarcinoma, or via hormonal disturbances during pregnancy [1,2]. Consequently, these conditions lead to bile acid accumulation in hepatic tissues causing hepatotoxicity [3,4].

Pathophysiologically, obstructive cholestasis increases biliary pressure that leads to rupture in cholangioles leading to the bile reflux back into hepatic tissues causing hepatotoxicity [5]. Hepatotoxicity initiates inflammatory response via secretion of osteopontin and CXC chemokines by hepatocytes which in turn leads to an extensive neutrophil accumulation that induces liver injury [6–11].

Lithocholic acid (LCA) is one of the bile acids that essentially acts as a detergent for dietary fat solubilization and absorption [12,13]. LCA is a secondary bile acid formed by colon bacterial enzyme called 7 α-dehydroxylase [14]. LCA is a hydrophobic compound implicated in several diseases such as colon cancer, hepatotoxicity, and liver injury [15,16]. Experimentally, LCA feeding was used as a model of liver injury by Fickert et al. [5] in which LCA precipitates in both hepatic and biliary tissues causing obstructive cholestasis and initiates the inflammatory cycle.

On the other hand, artemisinin is a chemical compound synthesized either naturally by a plant called *Artemisia annua*, or artificially [17]. It is a sesquiterpene lactone containing a peroxide bridge that might be responsible for its action [18].

Both artemisinin and its derivatives have been reported to be an effective treatment for several viral infections, toxoplasmosis, and against *Pneumocystis carinii*, as well as these compounds have been shown to be effective against some human cancer cell lines [19–21]. Furthermore, artemisinin has been shown to be a good treatment for different parasitic diseases such as malaria, leishmaniasis, and African sleeping sickness [22–25]. In the same context, artemisinin also has an anti-inflammatory and immunomodulatory effect [26]. A dosage of artemisinin between 100 and 1000 mg per day was rated possibly safe by WHO. In terms of liver injury, Chin et al. [27] have reported that dihydroartemisinin prevents liver fibrosis due to its action on both the apoptosis pathway and PDGF/MAPK pathway in experimental animals [28]. Other reports revealed that artemisinin and its derivatives help in regeneration of hepatic granulomatous lesions in experimental *Schistosoma mansoni* compared with the infected untreated group [29,30]. Thus, the objective of this project is to study the effect of artemisinin on LCA-induced liver injury in the animal model on both cellular and molecular scales.

## Materials and methods

### Materials

Adult male mice were purchased from King Fahd Institute for Scientific Research, King Abdulaziz University, Saudi Arabia. LCA was from Santa Cruz Biotechnology, Heidelberg Germany. Artemisinin extract was purchased from Doctor’s Best, Inc., California, U.S.A. Biochemical kits for liver and other profiles were from SOMATCO, JEDDAH, Prince Abdulaziz Ibn Musaid Ibn Jalawi.

### Animals and experimental procedure

Forty adult male mice, 8 weeks old, weighing 20–25 g were housed under conditions of controlled temperature (25 ± 2°C) with a 12-h day-night cycle in Medical Laboratory Department, College of Applied Medical Science, Turabah, Taif University. Animals gained free access to food and water *ad libitum*. All procedures were approved by the Animal Care Committee of Taif University.

#### Induction of cholestasis in mice and experimental design

Cholestatic groups were fed on 1% LCA mixed into the control diet and allowed food and water *ad libitum* for 0–96 h [31]. The present study was carried out on 40 adult healthy mice that were divided into 4 groups, 10 mice each; the negative control group didn’t receive any medication and gained free access to food and water. The normal group was fed normally with 100 mg/kg of artemisinin extract by oral gavage. The artemisinin dose was confirmed using HPLC. The cholestatic group fed on 1% LCA mixed into control diet and cholestatic group co-treated with 100 mg/kg of artemisinin extract by oral gavage. Mice were treated for 1 month then killed at end of the experiment.

##### Assay of biochemical parameters

Serum samples were analyzed by standard enzymatic assays using commercial kits for alanine aminotransferase (ALT), aspartate aminotransferase (AST), direct bilirubin (DBIL) and total bilirubin (TBIL), serum amylase in accordance with the manufacturer’s protocols (SOMATCO).

##### Gene expression and reverse transcription PCR

###### RNA extraction

For the preparation of total RNA, hepatic tissue samples (approximately 100 mg each) were collected from mice, flash frozen in liquid nitrogen and subsequently stored at −70°C in 1 ml Qiazol. Frozen samples were homogenized using a Polytron 300 D homogenizer (Brinkman Instruments, Westbury, NY). Then 0.3 ml chloroform was added to the homogenate. The mixture was shaken for 30 s followed by centrifugation at 4°C and 12500 rpm for 20 min. The supernatant layer was collected to a new set of tubes and an equal volume of isopropanol was added to the samples, shacked for 15 s and centrifuged at 4°C and 12500 rpm for 15 min. The RNA pellets were washed with 70% ethanol, briefly dried up, then dissolved in diethylpyrocarbonate (DEPC) water. The prepared RNA integrity was checked by electrophoresis. RNA concentration and purity were determined spectrophotometrically at 260 nm.

###### cDNA synthesis

For cDNA synthesis, the mixture of 2 µg total RNA and 0.5 ng oligo dT primer in a total volume of 11 µl sterilized DEPC water was incubated in the PeX 0.5 thermal Cycler (PCR machine) at 65°C for 10 min for denaturation. Then, 4 µl of 5× RT-buffer, 2 µl of 10 mM dNTPs and 100 U Moloney Murine Leukemia Virus (M-MuLV) Reverse Transcriptase was added in a total volume of 20 µl by DEPC water. The mixture was incubated again in the thermal Cycler at 37°C for 1 h, then at 90°C for 10 min to inactivate the enzyme.

###### Semi-quantitative PCR analysis

Specific primers for genes of tissue samples were designed using an Oligo-4 computer program and synthesized by Macrogen (Macrogen Company, GAsa-dong and Geumcheon-gu, Korea) listed in Table 1. PCR was conducted in a final volume of 25 µl consisting of 1 µl cDNA, 1 µl of 10 picomolar of each primer (forward and reverse) and 12.5 µl PCR master mix (Promega Corporation, Madison, WI), and the volume was brought up to 25 µl using sterilized, deionized water. The cycle sequence of PCR reaction was carried out at 94°C for 5 min one cycle, followed by 30–35 cycles each, which consisted of denaturation at 94°C for 1 min, annealing at the specific temperature corresponding to each primer (information about primer annealing temperature was outlined after primer design) and extension at 72°C for 1 min with additional final extension at 72°C for 5 min. As a reference, expression of glyceraldehyde-3-phosphate dehydrogenase (*G3PDH*) mRNA as a housekeeping gene was expressed. PCR products were electrophorized on 1% agarose gel stained with ethidium bromide in TBE (Tris-Borate-EDTA) buffer. PCR products were visualized under UV light and photographed using gel documentation system.

**Table 1:** PCR conditions for the genes analyzed

Gene	Product size	Annealing temperature	Sense	Antisense
***G3PDH***	269	59	tgttcctacccccaatgtgt	tgtgagggagatgctcagtg
***CYP2B10***	340	59	agtacccccatgttgcagag	ggaggatggacgtgaagaaa
***UGT1A1***	344	60.5	cctatgggtcacttgccact	cgatggtctagttccggtgt
***SULT2A1***	342	58.4	tcggctggaatcctaagaga	tgggaagatgggaggttatg
***CAR***	476	60.5	gggcttcttcagacgaacag	tctggtcctccatggttagg
***FXR***	483	59	agttgccgtgaggaagctaa	gtgagcgcgttgtagtggta
***ABCG8***	446	59	tctccaggtcctgattggtc	ggcaatcagagtcaacagca
***MRP2***	499	59	tcctagacagcggcaagatt	ctctggctgtccaacactca
***BSEP***	387	60.5	cctcagtgctttccttctgg	acagccacagagagggagaa
***OATP2***	358	58	acccaagaggctgtctctca	gccaacagaaatgccttgat

Abbreviations: *ABCG8*, ATP-binding cassette sub-family G member 8; *BSEP*, bile salt export pump; *CAR*, constitutive androstane receptor; *CYP2B10*, cytochrome P450 family 2 subfamily b, polypeptide 10; *FXR*, farnesoid x receptor; *MRP2*, multidrug resistance-associated protein 2; *OATP2*, organic anion-transporting polypeptide; *UGT1A1*, UDP glucuronosyltransferase family 1 member A1; *SULT2A1*, sulfotransferase family 2A.

##### Histopathological examination

The collected specimens of the liver from the killed mice were fixed in 10% buffered neutral formalin solution for at least 24 h and then routinely processed. Paraffin sections of 5 μ thickness were prepared, stained with hematoxylin and eosin stain (H&E) and then examined microscopically.

##### Immunohistochemical examination of glutathione and NFκB

Hepatic tissues were fixed in 10% buffered neutral formalin, washed, dehydrated, cleared, embedded in paraffin, cast then sectioned. Tissue sections were deparaffinized and treated with 3% H_2_O_2_ for 10 min to inactivate the peroxidases. Subsequently, samples were heated in 10 mM citrate buffer at 121°C for 30 min for antigen retrieval and blocked in 5% normal serum for 20 min, and pancreas was incubated with a rabbit polyclonal anti-glutathione primary antibody (1:100; sc-71155; Santa Cruz Biotechnology, Inc., Dallas, TX) or NFκB p50 antibody (1:100; sc-7178; Santa Cruz Biotechnology, Inc.) in PBS overnight at 4°C. After three extensive washes with PBS, the sections were incubated with a goat anti-rabbit IgG biotin-conjugated secondary antibody (1:2,000; sc 2040; Santa Cruz Biotechnology, Inc.) for 20 min at 32°C. After further incubation with horseradish peroxidase-labeled streptavidin, antibody binding was visualized using diaminobenzidine, and the sections were counterstained with hematoxylin.

### Statistical analysis

Results are shown as means ± standard error of means (SEM). Data analysis were done using ANOVA and *post hoc* descriptive tests by SPSS software version 11.5 for Windows (SPSS, IBM, Chicago, IL, U.S.A.) with *P*<0.05 considered as statistically significant. Regression analysis was done using the same software.

## Results

### Artemisinin prevented liver injury caused by LCA toxicity due to liver function tests

Data shown in Table 2 clearly demonstrated that LCA caused a significant increase in serum levels of both AST and ALT, which indicated a severe liver injury. Similarly, both direct and TBIL were significantly increased in mice exposed to LCA and that increase was accompanied by a significant reduction of serum levels of amylase as shown in Table 2. However, these changes were significantly ameliorated in LCA + artemisinin co-treated mice as shown in Table 2.

**Table 2:** Biochemical measurements of liver functions for normal control, artemisinin administrated mice, LCA, and LCA + artemisinin co-treated mice

Group	Parameter					
	AST (U/l)	ALT (U/l)	ALP (U/l)	TBIL (mg/dl)	DBIL (mg/dl)	Amylase (U/l)
Control	170 ± 1.62	49 ± 1.22	89 ± 1.06	0.03 ± 0.01	0.02 ± 0.02	3149 ± 131
Artemisinin	290 ± 43	58 ± 4.2	63 ± 8.8	0.18 ± 0.03	0.12 ± 0.04	2556 ± 177
Lithocholic A	5718 ± 367^†^	1665 ± 33.2^†^	61 ± 6.6	0.93 ± 0.03^†^	0.57 ± 0.04^†^	1853 ± 59^†^
LCA + artemisinin	179 ± 4.1*	42 ± 1.7*	63 ± 3.81	0.08 ± 0.02*	0.12 ± 0.03*	3160 ± 95*

Values are represented by mean ± SEM for triplicates experiments. ^†^Represents *P* values of LCA-treated mice corresponding to normal control. *Represents *P* values of LCA + artemisinin co-treated mice corresponding to LCA-treated mice.

### Histopathological changes in cholestatic mice and cholestatic mice co-treated with artemisinin extract

**Figure 1:**
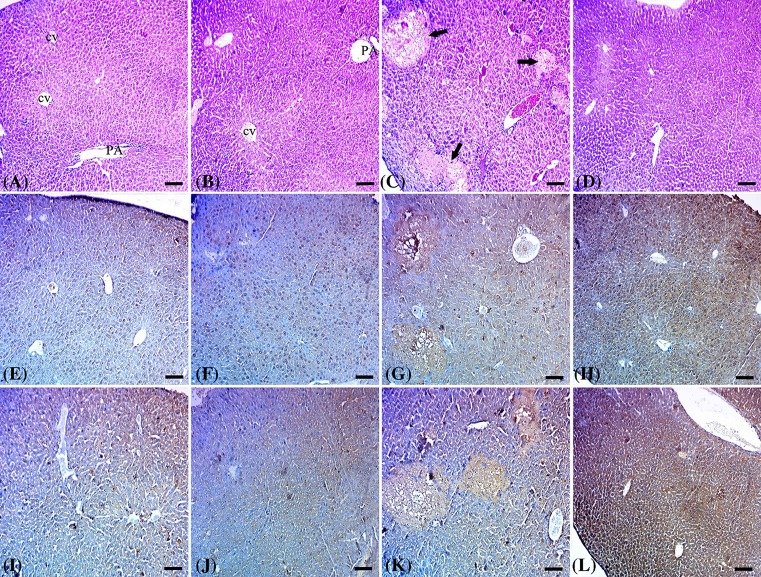
Results of histopathological and immunohistochemical examination. (**A** and **B**) livers of control and artemisinin groups respectively with normal tissue architecture. (**C**) Liver of LCA group with multiple necrotic foci of different sizes (arrows). (**D**) Liver of LCA group treated with Artemisinin showed healing of hepatic tissue. (**E** and **F**) Livers of control and artemisinin groups respectively with mild glutathione expression in hepatic tisuue. (**G**) Liver of LCA group showed high expression of glutathione in the necrotic foci and surrounding hepatic tissue. (**H**) Liver of LCA group treated with artemisinin showed strong expression of glutathione all over the hepatic tissue. (**I** and **J**) Livers of control and artemisinin groups respectively showed mild expression of NFκB in hepatic tissue. (**K**) Liver of LCA group showed high expression of NFκB in the necrotic foci with mild expression of surrounding hepatic tissue. (**L**) Liver of LCA group treated with artemisinin showed strong expression of NFκB all over the hepatic tissue (scale bar = 100 µm).

Hepatic tissues of control and artemisinin groups showed normal hepatic architecture with normal central veins, hepatic lobules, and hepatic sinusoids (Figure 1A,B respectively). Hepatic tissues of LCA group showed severe hepatotoxicity with multiple areas of necrosis of irregular distribution with an absence of both tissue architecture and cellular details (Figure 1C). Hepatic tissues of LCA group co-treated with artemisinin showed regeneration of hepatic lesions with mostly normal hepatic tissue (Figure 1D).

### Immunohistochemical changes of glutathione and NFκB in cholestatic mice and cholestatic mice co-treated with artemisinin extract

Hepatic tissues of control and artemisinin groups showed mild expression of glutathione in hepatic tissue (Figure 1E,F respectively). However, Hepatic tissues of the LCA group showed high expression of glutathione in the necrotic foci and surrounding hepatic tissue (Figure 1G). Hepatic tissues of the LCA group that was treated with artemisinin showed strong expression of glutathione all over the hepatic tissue (Figure 1H).

Hepatic tissues of control and artemisinin-treated animals showed mild expression of NFκB (Figure 1I,J respectively). Hepatic tissues of LCA administrated group showed high expression of NFκB in the necrotic foci with a mild expression of surrounding tissues (Figure 1K). Liver of LCA group that co-treated with artemisinin showed strong expression of NFκB all over the hepatic tissue (Figure 1L).

#### Molecular changes of multidrug resistance-associated protein 2, constitutive androstane receptor, and farnesoid x receptor expressions in cholestatic mice treated with artemisinin

**Figure 2:**
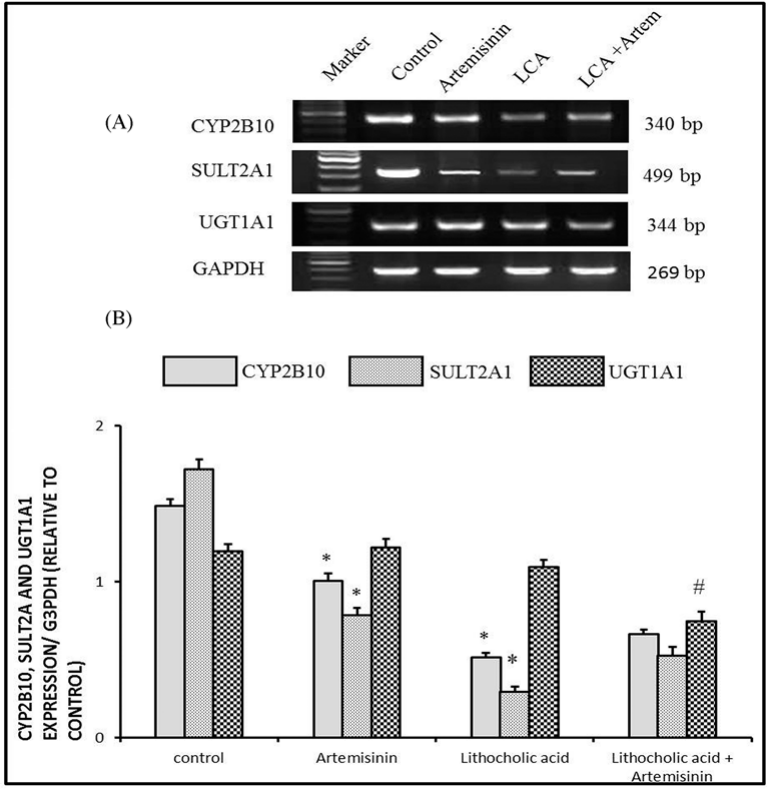
Effect of artemisinin on changes in mRNA expressions of MRP2, CAR, and FXR genes induced by LCA toxicity in liver with GAPDH as representative lanes Values are means ± SE of ten mice. *P<0.05 corresponding to control group; ^#^*P*<0.05 corresponding to LCA toxicity exposed group. Upper panels are mRNA expressions of examined genes. Lower columns are densitometric analysis of gene expression.

As presented in Figure 2, LCA model of cholestasis showed a significant down-regulation (*P*<0.05) in mRNA expressions of multidrug resistance-associated protein 2 (MRP2), constitutive androstane receptor (CAR), and farnesoid x receptor (FXR) compared with the control group. Cholestatic mice co-treated with artemisinin revealed a significant increase in expressions of previous genes (*P*<0.05).

#### Molecular changes of cytochrome P450 family 2 subfamily b, sulfotransferase family 2A, and UDP glucuronosyltransferase family 1 member A1expressions in cholestatic mice treated with artemisinin

**Figure 3:**
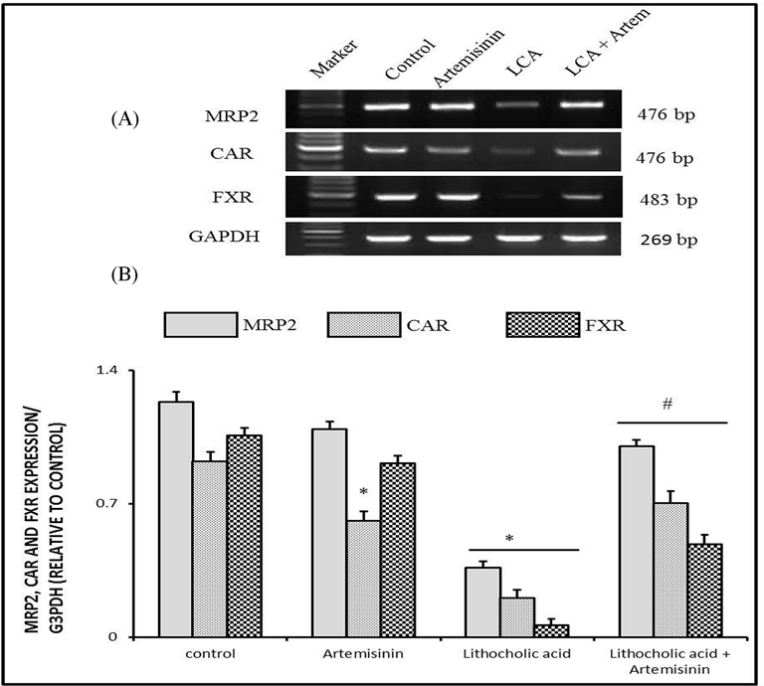
Effect of artemisinin on changes in mRNA expressions of *CYP2B10, SULT2A*, and *UGT1A1* genes induced by LCA toxicity in liver with *GAPDH* as representative lanes Values are means ± SE of ten mice. *P<0.05 corresponding to control group; ^#^*P*<0.05 corresponding to LCA exposed group. Upper panels are mRNA expression of examined genes. Lower columns are densitometric analysis of gene expression.

Regarding expressions of hepatic bile acid and bilirubin-metabolizing/detoxifying enzymes (cytochrome P450 family 2 subfamily b [*CYP2B10*], sulfotransferase family 2A [*SULT2A1*], and UDP glucuronosyltransferase family 1 member A1 [*UGT1A1*]), Figure 3 showed a significant decrease (*P*<0.05) in mRNA expressions of *CYP2B10* and *SULT2A1* in cholestatic mice compared with control group, while the expression of *UGT1A1* revealed no change in the LCA model of cholestasis. Treatment cholestatic mice with artemisinin restore *SULT2A1* mRNA expression significantly (*P*<0.05). However, there was no change in *CYP2B10* expression in mice co-treated with artemisinin.

#### Molecular changes of bile salt export pump, ATP-binding cassette sub-family G member 8, and organic anion-transporting polypeptide expressions in cholestatic mice treated with artemisinin

**Figure 4:**
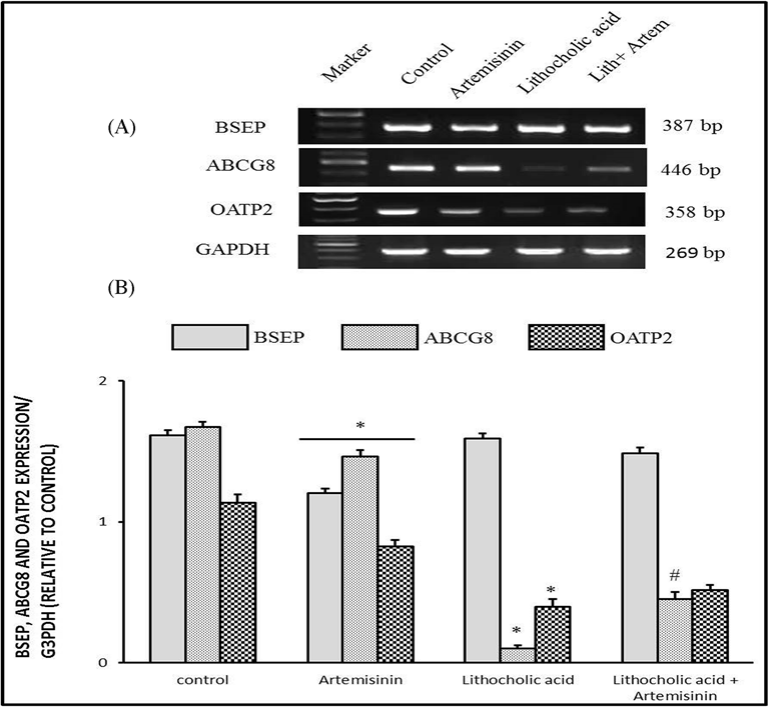
Effect of artemisinin on changes in mRNA expressions of *BSEP, ABCG8*, and *OATP2* genes induced by LCA toxicity in liver with *GAPDH* as representative lanes Values are means ± SE of ten mice. *P<0.05 corresponding to control group; ^#^*P*<0.05 corresponding to LCA exposed group. Upper panels are mRNA expression of examined genes. Lower columns are densitometric analysis of gene expression.

In cholestatic mice, there was a significant down-regulation (*P*<0.05) in hepatic mRNA expressions of ATP-binding cassette sub-family G member 8 (*ABCG8*) and organic anion-transporting polypeptide (*OATP2*) genes as shown in Figure 4 as compared with the control group. Bile salt export pump (*BSEP*) gene expression was not changed in cholestatic mice as compared with control group. Cholestatic mice co-treated with artemisinin showed a partial increase in expression of *ABCG8* gene as well as treatment with artemisinin had no effect on down-regulated expression of *Oatp2* gene.

#### Molecular changes of Oatp4 expressions in cholestatic mice treated with artemisinin

**Figure 5:**
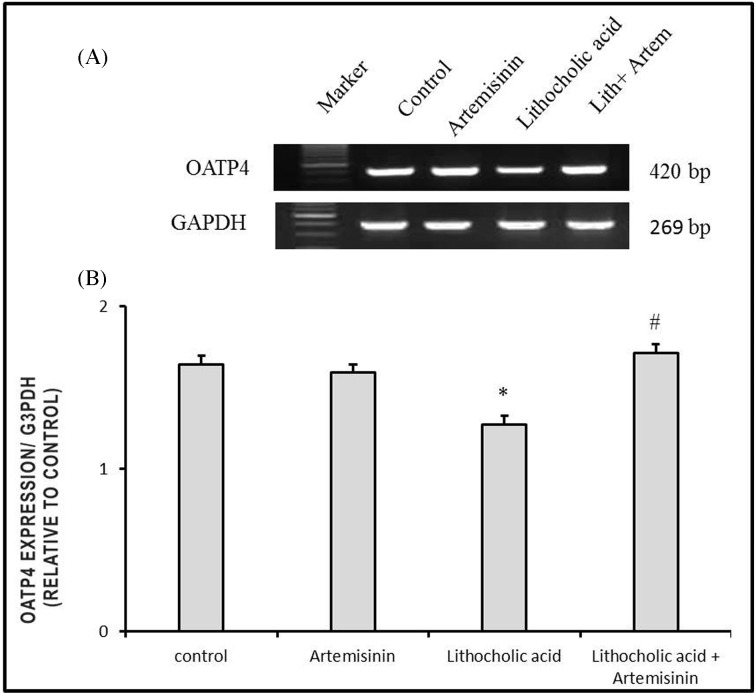
Effect of artemisinin on changes in mRNA expression of *OATP4* gene induced by LCA in liver with *GAPDH* as representative lanes. Values are means ± SE of ten mice. *P<0.05 corresponding to control group; ^#^*P*<0.05 corresponding to LCA-exposed group. Upper panels are mRNA expression of examined gene. Lower columns are densitometric analysis of gene expression.

Figure 5 demonstrated a significant decrease (*P*<0.05) of hepatic mRNA expressions of *Oatp4* in LCA model of cholestasis as compared with control mice. Significant restoration of *Oatp4* expression in cholestatic mice that co-treated with artemisinin.

## Discussion

Artemisinin is an effective natural treatment for several viral infections, toxoplasmosis and Pneumocystis carinii, and has been shown to be effective against some human cancer cell lines [19–21]. The present study clearly demonstrated that artemisinin had the potential to protect against LCA-induced liver cholestasis, as evidenced by increasing survival rate and ameliorating liver morphology and histology, as well as decreasing serum ALT/AST/ALP, serum total bile acids, TBIL, and amylase.

Cholestatic liver disease arises when the excretion of bile acids from the liver is interrupted. Bile acids, mainly produced from cholesterol in the liver, are required for the absorption and excretion of lipophilic metabolites such as cholesterol [32,33]. The excess accumulation of bile acids markedly alters the expression of various genes involved in cholesterol and phospholipid homeostasis resulting in severe liver injury represented by cell death and inflammation [34].

Furthermore, LCA is a hydrophobic secondary bile acid that is primarily formed in the intestine by the bacterial metabolism of chenodeoxycholic acid. Administration of LCA and its conjugates to rodents is known to cause intrahepatic cholestasis [35,36]. Cholestasis, functionally defined as a cessation or impairment of bile flow, can cause nutritional imbalance related to malabsorption of lipids and fat-soluble vitamins with severe liver damage as a result of the accumulation of toxins normally excreted in bile [37]. The potentially harmful effects of LCA and other bile acids are ameliorated by two hepatic detoxification pathways, namely hydroxylation and conjugation. These reactions make the bile acid more hydrophilic and facilitate its excretion in the feces or urine. Varieties of metabolic enzymes and transporters play crucial roles in bile acid homeostasis [38].

In the current study, artemisinin had a moderate impact on key metabolizing enzyme genes: *Cyp2b10* and *Ugt1a1*. Expression of *Cyp2b10* is thought to be mediated by *CAR* activation [39]. So, activation of *CAR* when cholestatic mice were treated with artemisinin led to moderate activation of *Cyp2b10.* However, artemisinin induced significant restoration of *Sult2a1* expression. Such activation is an important mechanism that aids in bile acid elimination [40].

When the excretion of bile acids is disrupted by disease, bile acids accumulate in hepatocytes, resulting in cholestasis. Once bile acid concentrations exceed their critical micellar concentration, they no longer aggregate with phospholipids as micelles. At that point the hydrophobic properties of bile acids are cytotoxic, leading to apoptotic or necrotic cell death. Excess concentrations of bile acids also cause adaptive changes in the liver, such as decreased hepatobiliary transport [41]. Moreover, Fickert et al. [42] have shown that administration of LCA for 4 days in mice causes hepatocellular necrosis with significant reductions in basolateral bile acid uptake. These adaptive changes in the liver represent an attempt to protect cells from the inherent toxicity of accumulating bile acids. Interestingly, Yu et al. [43] have reported that LCA is an *FXR* antagonist that is activated when treated with artemisinin to increase the Bsep expression and facilitates bile acid excretion. Therefore, down-regulation of a bile acid efflux transporter, such as *Bsep*, by LCA might help to explain why this monohydroxylated bile acid is considered one of the most toxic bile acid species.

Pharmacological activation of the *CAR* protects the liver when cholestasis is treated with artemisinin. The current study evaluates how activation of *CAR* influences genes involved in bile acid biosynthesis as a mechanism of hepatoprotection during bile acid-induced liver injury.

Expression of bile acid synthesis and detoxication enzymes are tightly regulated by nuclear hormone receptors and other transcription factors. One such nuclear receptor is *CAR. CAR* assists in the regulation of bile acid metabolism by inducing phase I and II enzymes, as well as bile acid transport proteins [44]. In addition, *SULT2A1* adds a sulfate moiety to LCA to increase its water solubility and subsequent excretion [45]. Previous studies have shown that pretreatment of mice with *CAR* activators protects against the hepatotoxicity of LCA-induced cholestasis [46].

Furthermore, we examined the effects of LCA and artemisinin on the expression of other genes involved in the transport and metabolism of bile acids, including those of *Mrp2* and the Na-independent *Oatp2*. LCA induced significant down-regulation in *Mrp2* and *Oatp* hepatic expressions. However, artemisinin treatment strongly induced increased *Oatp2* expression in the cholestatic mice. *Oatp2* is a basolateral (sinusoidal) transporter that can mediate hepatocellular uptake of a wide range of amphipathic substrates, including bile acids and xenobiotics [47,48]. Interestingly, like *Mrp2*, the basal level of *Oatp2* expression was increased in the cholestatic mice treated with artemisinin. Similarly, *Bsep*, as an essential transporter mediating canalicular bile acid output was slightly changed by LCA and artemisinin treatment slightly down-regulated its expression.

During the induction of cholestasis, *Mrp* transporters including *Mrp2, Mrp3*, and *Mrp4* exert their effects in favoring output of bile acid or bilirubin conjugated with glucuronide or sulfate [49]. In the present study, LCA significantly induced a decrease in the expression of *Mrp2* at mRNA level, but cholestatic mice treated with artemisinin revealed an increase in *Mrp2* expression significantly that may contribute to the hepatoprotection of artemisinin by enhancing bile acid output.

Histopathological findings clarify that oral exposure to LCA causes severe hepatotoxicity with multiple areas of necrosis of irregular distribution (Figure 1C). However, pictures of livers of LCA group co-treated with artemisinin showed normal hepatic architecture with normal central veins, hepatic lobules, and hepatic sinusoids (Figure 1D). Furthermore, both glutathione, as body antioxidant defense and NFκB were highly expressed in the necrotic foci and surrounding hepatic tissue in LCA-exposed animals compared with normal control group as shown by immunohistochemical staining (Figure 1G,K). These outcomes validate that LCA oral administration certainly causes liver injury leading to acute cholestasis. Treatment with artemisinin led to increasing expression of glutathione all over the hepatic tissue, thus acting as a natural antioxidant herb. Activation of NFκB in the treated group could be attributed to its role in activating genes related to cell survival or cellular proliferation.

In conclusion, the current study strikingly revealed that artemisinin extract treatment can prevent severe hepatotoxicity and cholestasis via LCA exposure and thus could be used as a treatment choice.

## Abbreviations

*ABCG8*: ATP-binding cassette sub-family G member 8
AST: aspartate aminotransferase
ALT: alanine aminotransferase
*CAR*: constitutive androstane receptor
*CYP2B10*: cytochrome P450 family 2 subfamily b
*BSEP*: bile salt export pump
DBIL: direct bilirubin
DEPC: diethylpyrocarbonate
*FXR*: farnesoid x receptor
*G3PDH*: glyceraldehyde-3-phosphate dehydrogenase
LCA: lithocholic acid
*MRP2*: multidrug resistance-associated protein 2
*OATP2*: organic anion-transporting polypeptide
*SULT2A1*: sulfotransferase family 2A
TBIL: total bilirubin
*UGT1A1*: UDP glucuronosyltransferase family 1 member A1
